# Parsimonious reconstruction of network evolution

**DOI:** 10.1186/1748-7188-7-25

**Published:** 2012-09-19

**Authors:** Rob Patro, Emre Sefer, Justin Malin, Guillaume Marçais, Saket Navlakha, Carl Kingsford

**Affiliations:** 1 Center for Bioinformatics and Computational Biology, University of Maryland, College Park, MD 20742, USA; 2 Department of Computer Science, University of Maryland, College Park, MD 20742, USA; 3 Computational Biology, Bioinformatics and Genomics Concentration, Biological Sciences Graduate Program, University of Maryland, College Park, MD 20742, USA; 4 Program in Applied Mathematics, Statistics, and Scientific Computation, University of Maryland, College Park, MD 20742, USA; 5 School of Computer Science, Carnegie Mellon University, Pittsburgh, PA 15213, USA

**Keywords:** Network evolution, Arsimony, Ancestral network reconstruction, Interaction networks, Regulatory networks

## Abstract

**Background:**

Understanding the evolution of biological networks can provide insight into how their modular structure arises and how they are affected by environmental changes. One approach to studying the evolution of these networks is to reconstruct plausible common ancestors of present-day networks, allowing us to analyze how the topological properties change over time and to posit mechanisms that drive the networks’ evolution. Further, putative ancestral networks can be used to help solve other difficult problems in computational biology, such as network alignment.

**Results:**

We introduce a combinatorial framework for encoding network histories, and we give a fast procedure that, given a set of gene duplication histories, in practice finds network histories with close to the minimum number of interaction gain or loss events to explain the observed present-day networks. In contrast to previous studies, our method does not require knowing the relative ordering of unrelated duplication events. Results on simulated histories and real biological networks both suggest that common ancestral networks can be accurately reconstructed using this parsimony approach. A software package implementing our method is available under the Apache 2.0 license at http://cbcb.umd.edu/kingsford-group/parana.

**Conclusions:**

Our parsimony-based approach to ancestral network reconstruction is both efficient and accurate. We show that considering a larger set of potential ancestral interactions by not assuming a relative ordering of unrelated duplication events can lead to improved ancestral network inference.

## Background

High-throughput experiments have revealed thousands of regulatory and protein-protein interactions that occur in the cells of present-day species. To understand why these interactions take place, it is necessary to view them from an evolutionary perspective. In analogy with ancestral genome reconstruction [1], we consider the problem of predicting the topology of the common ancestor of pathways, complexes, or regulatory programs present in multiple extant species.

Reconstructing plausible ancestral networks can help answer many natural questions about how present-day networks have evolved. For example, joint histories can be used to compare the conservation and the route to divergence of corresponding processes in two species. This allows us to more finely quantify how modularity has changed over time [2] and how interactions within a protein complex may have reconfigured across species starting from a single shared state [3]. Such analysis can also be integrated to develop better network alignment algorithms and better network-based phylogenies [4-8], and it can be used to study robustness and evolvability [9-11]. Further, inferred changes in metabolic networks can be linked to changes in the biochemical environment in which each species has evolved, and this can reveal novel mechanisms of ecological adaptation [12,13]. Finally, comparing network histories inferred using different model parameters can be used to estimate the likelihoods of various evolutionary events [14,15].

There has been some recent work on reconstructing ancestral interactions. Gibson and Goldberg [16] presented a framework for estimating ancestral protein interaction networks that handles gene duplication and interaction loss using gene trees reconciled against a species phylogeny. However, their approach assumes that interaction losses occur immediately after duplication and does not support interaction gain outside of gene duplication. These assumptions are limiting because interaction loses may occur well after duplication, and independent gains are believed to occur at non-trivial rates [17]. Dutkowski and Tiuryn [6] provided a probabilistic method for inferring ancestral interactions with the goal of improved network alignment. Their approach is based on constructing a Bayesian network with a tree topology where binary random variables represent existence or non-existence of potential interactions. A similar graphical model was proposed by Pinney et al. [18], who applied it to inferring ancestral interactions between bZIP proteins. In the former method, interaction addition and deletion is assumed to occur only immediately following a duplication or speciation event. Further, both methods assume the relative ordering of duplication events is known even between events in unrelated homology groups. Pinney et al. [18] also explore a parsimony-based approach [19] and find it to work well; however, it too assumes a known ordering of unrelated duplication events. The main drawback of these approaches is that the assumed ordering comes from sequence-derived branch lengths, which do not necessarily agree with rates that would be estimated based on network evolution [20]. This motivates an approach such as we describe below that does not use branch lengths as input.

Zhang and Moret [20,21] use a maximum-likelihood method to reconstruct ancestral regulatory networks as a means to improve estimation of regulatory networks in extant species. Mithani et al. [22] study the evolution of metabolic networks, but they only model the gain and loss of interactions amongst a fixed set of metabolites, whereas we also consider node duplication and loss encoded by a tree. Navlakha and Kingsford [15] present greedy algorithms for finding high-likelihood ancestral networks under several assumed models of network growth. They applied these methods to a yeast protein interaction network and a social network to estimate relative arrival times of nodes and interactions and found that the inferred histories matched many independently studied properties of network growth. This attests to the feasibility of using networks to study evolution. The authors, however, only consider a single network at a time, and there is no guarantee that independent reconstruction of two networks will converge to a common ancestor.

Here, we introduce a combinatorial framework for representing histories of network evolution that can encode gene duplication, gene loss, interaction gain and interaction loss at arbitrary times and does not assume a known total ordering of duplication events. We show that almost parsimonious histories of interaction gain and loss can be computed in practice quickly given a duplication history. In simulated settings, we show that these parsimonious histories can be used to accurately reconstruct a common ancestral regulatory network of two extant regulatory networks. We also show that our approach can infer, with high accuracy, the interactions among the bZIP family of proteins in several ancestral organisms.

## Methods

### A framework for representing network histories

Any natural model of network evolution will include events for gene duplication, gene loss, interaction gain, and interaction loss. Many such growth models have been studied (e.g. [9,21,23-26]). We describe below how these events can be encoded in a history graph. We note that there are other evolutionary events that affect the growth and structure of biological networks. For example, Toll-Riera et al. [27] provide evidence for *de novo* gene birth originating from non-coding genomic regions. While such events play a role in shaping the evolutionary history and current structure of biological networks; they are less common than the gene duplication and loss and interaction gain and loss, and are not explicitly modeled in the current framework.

Consider a set *V * of proteins or genes (henceforth “nodes”) descended from a common ancestor by duplication events. Those duplication events can be encoded in a binary *duplication treeT* with the items of *V * as the leaves. An internal node *u* in *T* represents a duplication event of *u* into its left and right children, *u*_*L*_ and *u*_*R*_. In this representation, after a duplication event, the node represented by *u* conceptually does not exist anymore and has been replaced by its two children. The leaves of a duplication tree are labeled *Present* or *Absent*. Absent leaves represent products of duplication events that were subsequently lost. A collection of such trees is a *duplication forest F*.

The gain and loss of interactions can be represented with additional non-tree edges placed on a duplication forest. A non-tree edge {*u*,*v*} represents an *edge flip event*, where the interaction between *u* and *v* is created if the interaction is currently absent or removed if the interaction is currently present. Let *P*_*u*_ and *P*_*v*_ be the paths from nodes *u* and *v* to the root. An interaction exists between *u* and *v* if there are an odd number of such flip non-tree edges between nodes in *P*_*u*_ and *P*_*v*_. Every non-tree edge between *P*_*u*_ and *P*_*v*_, therefore, represents alternatively interaction creation or deletion between nodes *u* and *v* in the evolution of the biological network.

**Figure 1:**
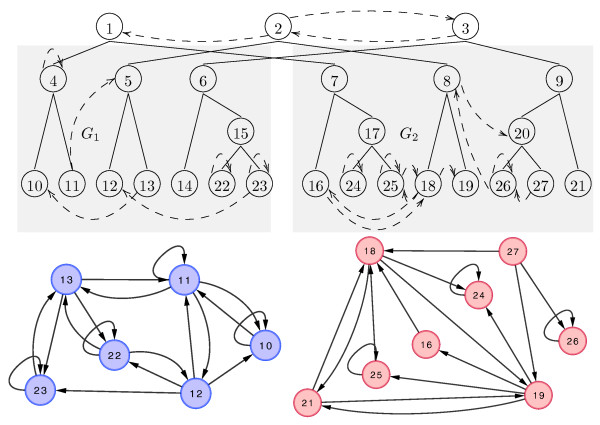
**Framework Overview.** A duplication forest (solid edges at top) with the non-tree edges (dashed) necessary to construct *G*_1_ and *G*_2_ (shown at bottom). Nodes 1, 2, and 3 represent the 3 homology groups present in the ancestral graph. Node 14 was lost. As an example of the connectivity induced by the non-tree edges, consider edge (27,18) in *G*_2_ which is implied by the directed non-tree edge from (3,2). However, the reverse edge (18,27), which is implied by (2,3), does not exist because its state is flipped by (8,20).

A graph *H* consisting of the union of a duplication forest and flip non-tree edges is a *network history*. A history *Hconstructs* a graph *G* when the Present leaves of the duplication forest in *H* correspond to the nodes of *G* and the flip edges of *H* imply an interaction between *u* and *v* if and only if {*u*,*v*} is an interaction in *G*. See Figure 1 for an example history.

Not all placements of non-tree edges lead to a valid network history. The interaction histories have to be consistent with some temporal embedding of the tree. Let $t_{u}^{c}$ and $t_{u}^{d}$ be respectively the time of creation and duplication of node *u*. Naturally, $t_{u}^{c}<t_{u}^{d}$, $t_{u}^{d}=\infty$ if *u* is a Present leaf, and if *v* is the child of *u*, then by definition we have

(1) $$t_{u}^{c}<t_{u}^{d}=t_{v}^{c}<t_{v}^{d}.$$

If {*u*,*w*} is a flip edge, then the time *t*_{*u*,*w*}_ of appearance of this edge must satisfy

(2) $$t_{u}^{c}\leq t_{\left\{u,w\right\}}<t_{u}^{d}\;\text{and}\;t_{w}^{c}\leq t_{\left\{u,w\right\}}<t_{w}^{d},$$

because an event between *u* and *w* can only occur when both *u* and *w* exist. A history graph *H* is said to be *valid* if there exist $t_{u}^{c},t_{u}^{d}$ for every node *u* such that conditions (1) and (2) are satisfied for every non-tree edge.

**Figure 2:**
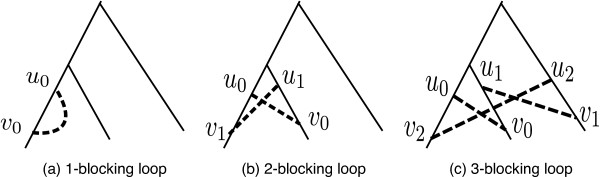
**Blocking Loops.** Blocking loops of size 1, 2 and 3. The solid lines represent a subset of the tree *T*. The dashed lines are non-tree edges representing interaction flip events.

Whether a particular history is valid can be checked combinatorially using the following alternative characterization of validity. A *k-blocking loop* is a set of flip edges {{*u*_*i*_,*v*_*i*_}}_0≤*i*<*k*_ such that *u*_*i* + 1_ is an ancestor of *v*_*i*_ in the tree for 0 ≤* i *<* k* (where the index *i* + 1 is taken modulo *k*). See Figure 2 for examples. Blocking loops are not permitted in valid histories and, conversely, the non-existence of blocking loops implies that a history is valid, as shown in Prop. 11.

#### Proposition 1

A history graph H is valid if and only if it does not have any blocking loop of any length.

#### Proof

Suppose there is a *k*-blocking loop. Using the same notation as above, we have the inequalities

(3) $$t_{u_{0}}^{d}>t_{\left\{u_{0},v_{0}\right\}}\geq t_{v_{0}}^{c}\geq t_{u_{1}}^{d}>t_{\left\{u_{1},v_{1}\right\}}\geq \ldots \geq t_{v_{k-1}}^{c}\geq t_{u_{0}}^{d},$$

which is a contradiction. Hence, to not have any blocking loops is necessary.

Conversely, suppose that *H* does not have any blocking loops. We assign times to the nodes and non-tree edges using a modified depth-first search (DFS) algorithm following the tree edges only. First, the root of the tree is given a creation time of 0. During DFS, just before calling DFS recursively on the left and right children of a node *u*, we set the duplication time $t_{u}^{d}=\max\{\max t_{\left\{u,v\right\}}+1,\overset{c}{\underset{u}{t}}+1\}$, where the second max is taken over all non-tree edges adjacent to *u*. Also, we set the creation time of the children $t_{u_{L}}^{c}=t_{u_{R}}^{c}=t_{u}^{d}$.

When DFS visits a node *u* with some non-tree edge {*u*,*v*} where *v* has not been assigned a creation time, *u* is added to a set *Q* and DFS is not called recursively on the children of *u*. The main loop consists of calling DFS again on all the nodes in *Q* until this set is empty. By construction, the algorithm assigns times which satisfy conditions (1) and (2). Therefore, if the algorithm terminates, *H* is a valid history.

At each main iteration, the nodes in the set *Q* are all the nodes *u* for which $t_{u}^{c}$ is set but $t_{u}^{d}$ is not set. It suffices to show that at each such iteration, at least one of the nodes in the set *Q* will not be added again to *Q* by a call to DFS. In other words, for at least one node *u *∈* Q*, every non-tree edge {*u*,*v*} has $t_{v}^{c}$ set. For a contradiction, suppose not. Take *u*_1_ ∈* Q*and {*u*_1_,*v*_1_} with $t_{v_{1}}^{c}$ not set. There is necessarily an ancestor of *v*_1_, call it *u*_2_, which is in *Q*. Similarly, take {*u*_2_,*v*_2_} with $t_{v_{2}}^{c}$ not set and its ancestor *u*_3_ ∈* Q*, and so on. Because *Q* is finite, *u*_*j *_=* u*_*i *_for some *j *>* i*, and we constructed a blocking loop. Hence, the algorithm must terminate. □

### Parsimonious reconstruction of a network history

Traditional phylogenetic inference algorithms and reconciliation between gene and species trees can be used to obtain duplication and speciation histories [28-30]. What remains is the reconstruction of interaction gain and loss events. This leads to the following problem:

#### Problem 1

(Minimum Flips) Given a duplication forest *F* and an extant network *G*, find *H*, a valid history constructing *G*, with a minimum number of flip edges.

We will show that nearly optimal solutions to this problem for a large range of instances can be solved in polynomial time in practice. Whether Problem 1 is NP-hard or admits a polynomial-time algorithm for all instances remains open.

#### A fast heuristic algorithm

The challenge of Problem 1 comes from avoiding the creation of blocking loops. A polynomial-time algorithm can find a minimum set of flip edges that reconstructs a graph *G* and does not contain 1- and 2-blocking loops but allows longer blocking loops. We define an *interaction encoding* of *G *= (*V*,*E*) as a function *f*_*G*_:* V *×* V *→ {0,1} such that *f*_*G *_(*u*,*v*) = 1 if {*u*,*v*} is an interaction in *G* and *f*_*G *_(*u*,*v*) = 0 otherwise. We omit the subscript on *f*_*G *_if *G* is clear from the context.

The following intertwined dynamic programming recurrences find the minimum number of flip edges required for *H* to construct a given graph *G* if blocking loops of length ≥ 3 are allowed. First, *S *(*u*,*f*) finds the minimum number of flip edges for the subtree rooted at *u* and interaction encoding *f *:

(4) $$S(u,f)=S(u_{L},f)+S(u_{R},f)+A(u_{L},u_{R},f).$$

The expression *A *(*u*,*v*,*f*) gives the minimum number of flip edges that should be placed between the subtree rooted at *u* and the subtree rooted at *v*. This can be computed using the recurrence:

(5) $$A(u,v,f)=\text{min}\left\{\begin{array}{c} A(u_{L},v,f)+A(u_{R},v,f)\;\\ A(u,v_{L},f)+A(u,v_{R},f)\;\\ 1+A(u_{L},v,\bar{f})+A(u_{R},v,\bar{f})\;\\ 1+A(u,v_{L},\bar{f})+A(u,v_{R},\bar{f}). \end{array}\right)$$

In the above, if one of *u* or *v* is a leaf but the other is not, the options that look at non-existent children are disallowed.

The function $\bar{f}$ in Eqn. (4) is defined as 1−*f* and thus represents a function such that $\bar{f}(x)$ has opposite parity from *f*(*x*) for all *x*. The *A* recurrence considers two possible options: (1) We connect *u* and *v* with a non-tree edge, this costs us 1 and flips the parity of all interactions going between the subtree rooted at *u* and the subtree rooted at *v*; or (2) We do not connect *u* and *v* with a flip edge. This costs 0 and keeps the parity requirement the same. Regardless of the choice to create an edge, because we are not allowed to have a 2-blocking loop, either (a) we possibly connect *u* to some descendant of *v* (and do not connect *v* to a descendant of *u*) or (b) we possibly connect *v* to some descendant of *u* (and do not connect *u* to a descendant of *v*).

The base case for the *S* recurrence when *u* is a leaf and the base case for the *A* recurrence when *u* and *v* are leaves are:

(6) S(u,f)=0andA(u,v,f)=f(u,v).

The minimum number of flip edges needed to turn a duplication forest *F* into a history constructing *G* (allowing blocking loops of ≥ 3) is then given by ∑_*r*_*S*(*r*,*d*_*G*_) + ∑_*r*,*q*_*A*(*r*,*q*,*d*_*G*_), where *d*_*G*_ is the interaction encoding of *G*, and the sums are over roots *r *,*q *of the trees in *F*. Standard backtracking can be used to recover the actual minimum edge set. If *n* is the number of nodes in the forest, the dynamic program runs in *O*(*n*^2^) time and space because only two functions *f * are ever considered: *d*_*G*_, and ${\bar{d}}_{G}$. This yields ≈* n *×* n *× 2 subproblems, each of which can be solved in constant time.

The heuristic also can be extended to handle different costs for interaction addition and deletion by changing the constants in the recurrences to be a function of the parity of each flip. Only two values of *f * (_*dG*_ and ${\bar{d}}_{G}$) are ever considered, and every flip switches *f * between these two states. Thus, by examining *f *, and determining if its current states corresponds to *d*_*G*_or ${\bar{d}}_{G}$, one can determine if an odd or even number of flips have occurred, and thus, whether the current flip corresponds to the addition or deletion of an interaction. If the current flip represents the addition of an interaction, then it incurs the cost *c*_add_. Otherwise, the flip encodes the loss of an interaction, and incurs the loss cost *c*_loss_.

#### Identifying and removing blocking loops

To identify blocking loops, we use a modified depth-first search procedure in which tree edges are traversed according to their direction (i.e away from the root) while non-tree edges can be traversed in either direction. Whenever a node is encountered twice during the depth first search, a cycle has been discovered and is checked for the blocking loop condition given above. If the cycle is not blocking loop, we can safely ignore it. Otherwise, one of the non-tree edges of this loop is chosen at random, and we forbid that edge from appearing in the solution and rerun the dynamic program. Because there are *O*(*n*^2^) possible non-tree edges, iterating this procedure will terminate in polynomial time. We repeat the process of identifying blocking loops and forbidding non-tree edges until a valid solution is obtained. In the worst case, one may obtain a solution where all non-tree edges are placed at leaves, but in practice long blocking loops do not often arise, and the obtained solutions are close to optimal (see section below).

#### Reconstruction of a common ancestor of two graphs

Given extant networks of several species, in addition to the reconstructed history, we seek a parsimonious estimate for their common ancestor network. Specifically, given extant networks *G*_1_ and *G*_2_, with interaction encodings *d*_1_ and *d*_2_, and their duplication forests *F*_1_ and *F*_2_, we want to find an ancestral network *X *= (*V*_*X*_,* E*_*X*_) such that the cost of *X* evolving into *G*_1_ and *G*_2_ after speciation is minimized. *V*_*X *_is the set of roots of the homology forests. We assume that the networks of the two species evolved independently after speciation. Therefore, we can use the recurrence above applied to *F*_1_ and *F*_2_ to compute $A_{F_{1}}(r,q,d_{1})$ and $A_{F_{2}}(r,q,d_{2})$ independently for *r*,*q *∈* V*_*X*_, and then select interactions in *X* as follows. *E*_*X*_ of *X* is given by the pairs *r*,* q *∈*V*_*X *_×* V*_*X *_ for which creating an interaction leads to a lower total cost than not creating an interaction. Formally, we place an interaction {*r*,*q*} in *E*_*X*_ if

(7) $$\;1\;+\;A_{F_{1}}(r,q,{\bar{d}}_{1})\;+\;A_{F_{2}}(r,q,{\bar{d}}_{2})<A_{F_{1}}(r,q,d_{1})\;+\;A_{F_{2}}(r,q,d_{2}).$$

Rule (5) creates an interaction in *X* if doing so causes the cost of parsimonious histories inferred for *G*_1_ and *G*_2_ between the homology groups associated with *r* and *q* to be smaller than if no interaction was created.

#### Modifications for self-loops

Self-loops (homodimers) can be accommodated by modifying recurrence (3):

(8) $$\;S^{\prime }(u,f)=\text{min}\left\{\begin{array}{c} S^{\prime }(u_{L},f)+S^{\prime }(u_{R},f)+A(u_{L},u_{R},f)\;\\ 1+S^{\prime }(u_{L},\bar{f})+S^{\prime }(u_{R},\bar{f})+A(u_{L},u_{R},\bar{f}). \end{array}\right)$$

The intuition here is that paying cost 1 to create a self-loop on node *u* creates (or removes) interactions, including self-loops, among all the descendants of *u*.

#### Modifications for directed graphs

The algorithm can be modified to handle evolutionary histories of directed graphs. For this, only the recurrence *A* need be modified. When computing *A*^*′ *^(*u*,*v*,*f*), a non-tree edge can be included from *u* to *v*, from *v* to *u*, both, or neither. Each of these cases modifies the function *f * in a different way. Specifically:

(9) $$A^{\prime }(u,v,f)=\text{min}\left\{\begin{array}{c} 0+A^{\prime }(u_{L},v,f)+A^{\prime }(u_{R},v,f)\;\;\;\;\\ 1+A^{\prime }(u_{L},v,\overset{\leftarrow }{f})+A^{\prime }(u_{R},v,\overset{\leftarrow }{f})\\ 1+A^{\prime }(u_{L},v,\overset{\to }{f})+A^{\prime }(u_{R},v,\overset{\to }{f})\\ 2+A^{\prime }(u_{L},v,\overset{↔}{f})+A^{\prime }(u_{R},v,\overset{↔}{f}),\\ \vdots \;\;\;\;\; \end{array}\right)$$

where the vertical ellipsis indicates the symmetric cases involving *v*_*L*_ and *v*_*R*_, and where $\overset{\to }{f},\overset{\leftarrow }{f},\overset{↔}{f}$ are defined, depending on *u* and *v*, as follows:

(10) $$\begin{array}{ll} \overset{\to }{f}(x,y) & =\text{min}\left\{\begin{array}{cc} 1-f(x,y) & \;\text{if}\;x\in \mathrm{ST}(u)\;\text{and}\;y\in \mathrm{ST}(v)\\ f(x,y) & \text{otherwise} \end{array}\right)\;\\ \overset{↔}{f}(x,y) & =\text{min}\left\{\begin{array}{cc} 1-f(x,y) & \;\text{if x}\in \text{ST (u) and}\\ & \;y\in \mathrm{ST}(v)\;\text{or vice versa}\\ f(x,y)\;\;\;\; & \text{otherwise}, \end{array}\right)\; \end{array}$$

with $\overset{\leftarrow }{f}$ defined analogously to $\overset{\to }{f}$. Here, ST(*u*) indicates the set of nodes in the subtree rooted at *u*.

### Accounting for phylogenetic branch lengths

One of the strengths of our proposed method is that it does not require the user to specify the lengths of the edges in a duplication history. The estimation of such phylogenetic branch lengths relies on the molecular clock assumption, and these lengths can easily be misestimated, especially those for distant ancestors.

However, previous approaches [18,19] relied crucially upon the phylogenetic branch lengths to impose a specific ordering on the set of potential ancestral interactions. Small errors in the estimates of phylogenetic branch lengths can lead these approaches to disallow potentially high probability or high parsimony ancestral interactions.

Yet, the branch lengths in the duplication history do encode potentially useful information. For example, two ancestral proteins for which the intervals of existence are separated by a significant amount of time are unlikely to have interacted, even if branch length estimates are imprecise. The algorithm we defined above can be further modified to account for branch lengths, using them to penalize unlikely ancestral states without explicitly disallowing potentially important interactions. This can be achieved by modifying the recurrence as follows:

(11) $$\;A(u,v,f)=\text{min}\left\{\begin{array}{c} A(u_{L},v,f)+A(u_{R},v,f)\;\;\;\\ A(u,v_{L},f)+A(u,v_{R},f)\;\;\;\\ \alpha \delta (u,v)+1+A(u_{L},v,\bar{f})+A(u_{R},v,\bar{f})\\ \alpha \delta (u,v)+1+A(u,v_{L},\bar{f})+A(u,v_{R},\bar{f}). \end{array}\right)$$

where

(12) $$\delta (u,v)=\left\{\begin{array}{cc} t_{v}^{c}-t_{u}^{d} & \;\text{if}\;t_{u}^{d}<t_{v}^{c}\\ t_{u}^{c}-t_{v}^{d} & \;\text{if}\;t_{v}^{d}<t_{u}^{c}\\ 0\;\; & \;\text{otherwise} \end{array}\right)$$

The analogous modification applies to the directed recurrence as well. Here, *α δ*(·,·) is a function that assigns a cost to a pair of nodes {*u*,*v*} that is proportional to the distance between the existence intervals of these nodes (and is 0 if they overlap). The constant, *α*, is provided as input to the algorithm and can be interpreted as the factor by which interactions are penalized between nodes which do not overlap in time according to the inferred phylogenetic branch lengths. At *α *= * ∞*, branch lengths become hard constraints, and proteins between which the existence intervals do not overlap are not allowed to interact; this *α*also prohibits the formation of blocking loops. However, results tend to be better (higher F1-score) when one allows some constraints from branch lengths to be violated. This approach allows our algorithm to take phylogenetic branch lengths into account in a way that incorporates the information they encode without suffering from the potential issues that occur when considering these lengths as hard constraints.

## Results and discussion

We analyze the performance of our parsimony-based approach to ancestral network reconstruction on both simulated and real biological data. To generate simulated data, we consider a number of plausible models of network evolution and show that the parsimony approach is able to reconstruct ancestral networks reasonably well over a wide range of model parameters. Further, following the experiment of Pinney et al. [18], we evaluate the performance of our approach on reconstructing the state of several ancestral network states of the bZIP family of proteins. We observe that our parsimony-based approach obtains high precision and recall, even on fairly distant ancestral networks.

### Generating plausible simulated histories

We use a *degree-dependent model* (DDM) to simulate the evolutionary path from a putative ancestral network to its extant state. The model simulates node duplication, node deletion, independent interaction gain, and independent interaction loss with given probabilities P_ndup_, P_nloss_, P_egain_ and P_eloss_, respectively. The nodes or edges involved in a modification are chosen probabilistically based on their degrees (as in [31]) according to the following expressions:

(13) $$\begin{array}{lll} & P(u|\text{node duplication})\propto 1/k_{u}\; & \;\\ & P(u|\text{node loss})\propto 1/k_{u}\; \end{array}$$

(14) $$\begin{array}{lll} & P((u,v)|\text{interaction gain})\propto k_{u}^{o}\; & \;\\ & P((u,v)|\text{interaction loss})\propto 1/k_{u}^{o},\; \end{array}$$

where $k_{u}^{o}$ is the out-degree of a node *u*, and *k*_*u *_is the total degree. At each time step, the distribution of possible modifications to the graph is calculated as P(modification) = P_operation_ P(object ∣ operation). Nodes with out-degree of 0 are removed. Varying parameters P_ndup_, P_nloss_, P_egain_ and P_eloss_ can produce a wide variety of densities and sizes. We also consider a *degree-independent model* (DIM) in which the four conditional probabilities in Eqns. (11) and (12) are all equal.

The DDM model is theoretically capable of producing evolutionary trajectories between any two networks while incorporating preferential attachment to the source node and random uniform choice of the target node. Furthermore, choosing a node for duplication or loss in inverse proportion to its degree favors an event in inverse relation to its expected disruption of the network.

We also consider a model of regulatory network evolution by Foster et al. [32], which is based on gene duplication, with incoming and outgoing interactions kept after duplication as in other models (P_inkeep_ and P_outkeep_ probabilities respectively). New edges are added with probability P_innovation_.

In all of the network evolution models, we started with a random connected seed graph that has 10 nodes and 25 interactions. We evolved it to *X* by 200 operations after which we introduce a speciation event, and then both *G*_1_ and *G*_2_ evolve from *X* by an additional 200 operations each. To generate more biologically plausible ancestral graphs, instances were kept only if the ancestral graph *X* had an in-degree that fit an exponential distribution with parameter between 1.0 and 1.2 or an out-degree that was scale-free with parameter between 1.8 and 2.2.

#### Reconstructing simulated networks

##### Optimality of loop breaking

**Figure 3:**
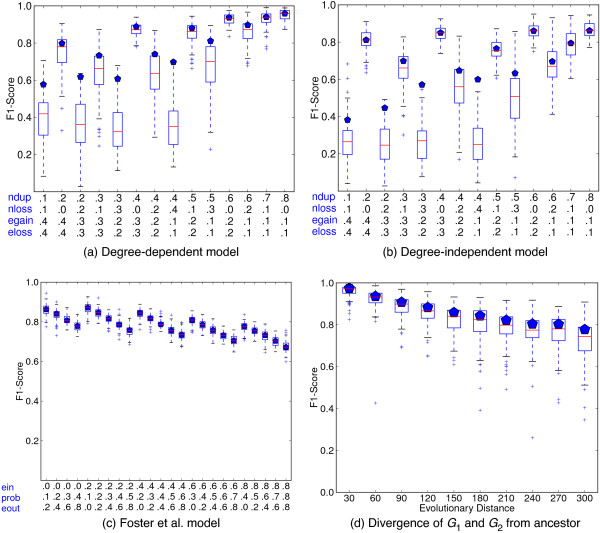
**Synthetic Performance.** (**a-c**) Effect of model parameters on reconstruction accuracy under three different models. “Prob” in (**c**) is P_innovation_. (**d**) Effect of evolutionary distance (number of network modification operations) on the quality of the ancestral network reconstruction. In both plots, boxes show 1st and 3rd quartile over 100 networks with median indicated by a line. Pentagons show the median if interactions incident to nodes lost in both lineages are not considered.

The greedy procedure to break blocking loops produces histories that are very close to optimal. We generated 1400 networks using the DDM model with the range of parameters shown on the x-axis of Figure 3a. In the vast majority of cases (1325 out of 1400), either no loop breaking is required, or the solution discovered after greedily breaking all loops has the same cost as the original solution. In these cases, therefore, the method returned a provably maximally parsimonious set of interaction modification events. In the remaining 75 cases (5.4*%*), greedily removing blocking loops increased the number of interaction modifications by no more than 10 (< 2*%* of the initial number of interaction modification events). Since the initial solution provides a lower bound on the optimal, we can verify that the greedy procedure always found a solution within 2% of the optimal (and perhaps even better). Thus, it seems that in practice, while blocking loops occur, the greedy procedure does a good job of eliminating them without increasing the number of events significantly.

##### Effect of growth model and its parameters

Modeling the evolutionary dynamics of a regulatory network is still an active topic of research. We therefore experimented with three different network models. Despite their differences, high precision and recall (implied from the F1 score) can be obtained for all of them for many choices of their parameters (Figure 3a-c). We measure the precision (defined as true positive/(true positive + false positive)), the recall (defined as true positive/(true positive + false negative)) and compute the F1-score (the harmonic mean of precision and recall: 2 · precision ·recall/(precision + recall)). Very good performance can be achieved under the general model presented above whether degree distributions are taken into account (Figure 3a) or not (Figure 3b) when selecting nodes and interactions to modify. In these cases, for most parameter choices, precision is close to 1.0, meaning every interaction predicted to be in the ancestor, in fact, was. Recall is often lower. The Foster et al. [32] model, with its heavy reliance on duplication events and lack of node loss events, tends to be the simplest under which to reconstruct the ancestral graph (Figure 3c).

The largest factor leading to poorer performance is lower recall caused by gene losses. If all descendants of a gene are lost in both extant networks, it is not possible to reconstruct interactions incident to it. If these interactions are excluded from the computation of recall, the F1 score often improves dramatically. Median F1 scores excluding these interactions are shown as pentagons in Figure 3.

##### Robustness to evolutionary divergence

Naturally, the ability to recover the ancestral network degrades as time passes and the extant networks diverge. However, the degradation is slow (Figure 3d, using the degree-dependent model with parameters fixed at P_ndup_ = 0.35, P_nloss_ = 0.05, P_egain_ = 0.3, and P_eloss_ = 0.3). When the distance is small (measured as the number of events separating them), we are almost always able to recover the ancestral network well, as illustrated by the high F1-scores and small interquartile ranges in Figure 3d. Even when the distance between the ancestral and extant networks is large (300) compared to the average ancestral network size (55), we obtain an F1-score of 0.72 (0.77 when homology groups lost in both lineages are not considered).

#### Reconstructing ancestral bZIP networks

We also repeated the test performed by Pinney et al. [18] by using our method to reconstruct ancestral interactions among the bZIP family of transcription factors. The interactions between dimerizing bZIP transcription factors are strongly mediated by their coiled-coil leucine zipper domains, and the strength of these interactions can be computationally predicted with high sensitivity and specificity using sequence alone [33]. This sequence-based method was used to predict both the interaction strength between extant bZIP proteins and inferred ancestral protein sequences. These interactions were used as the ground truth [18]. The duplication history relating the bZIP proteins is built atop the extant networks of 4 relatively distant species, *D. rerio*, *T. rubripes*, *H. sapiens*, and *C. intestinalis*. From the interactions in these extant networks and the structure of the duplication history of the constituent proteins, we reconstruct 3 ancestral networks: the Teleost (ancestor of *D. rerio* and *T. rubripes*), Vertebrata (ancestor of *D. reo*, *T. rubripes* and *H. sapiens*) and Chordate (ancestor of *D. rerio*, *T. rubripes*, *H. sapiens*, and *C. intestinalis*) networks.

Table 1 compares the relative performance of our parsimony-based approach and the probabilistic method described by Pinney et al. [18] Our results were generated using a ratio of 11.4 : 1 for the cost of interaction creation to interaction deletion (the same ratio as was used in the probabilistic method). Furthermore, we choose not to penalize interactions based on phylogenetic branch length (i.e. *α *= 0 in *δ*_*α*_), thus allowing our algorithm to explore the entire solution space. We note that our approach outperforms the probabilistic method, particularly on the Teleost and Vertebrata networks. One explanation for the improved performance of our method is that it considers a larger set of ancestral interactions by not explicitly disallowing parsimonious interactions based solely on potentially misleading phylogenetic branch lengths.

**Table 1:** bZIP Reconstruction Performance

**Ancestor**	**Method**	**Precision**	**Recall**	**F1**
Teleost	Parsimony	0.84	0.91	0.87
	Probabilistic	0.68	0.88	0.77
Vertebrata	Parsimony	0.79	0.94	0.86
	Probabilistic	0.75	0.81	0.78
Chordata	Parsimony	0.67	0.87	0.76
	Probabilistic	0.74	0.74	0.75

The relative performance of our parsimony approach and the probabilistic method described by Pinney et al. in reconstructing the ancestral interaction networks we consider.

We corroborated this hypothesis by measuring the reconstruction performance of our approach for increasing values of *α*, and noticed a very slow but steady decrease in performance as *α *increases. Nonetheless, at *α* = *∞*(using branch lengths as hard constraints as Pinney et al. do), our method still outperforms the probabilistic method on the Teleost network (F1 score of 0.84 vs 0.77). This experiment suggests that, at least on this family of protein interactions, relying on the phylogenetic branch lengths to aid inference does not improve — and potentially harms — performance.

**Figure 4:**
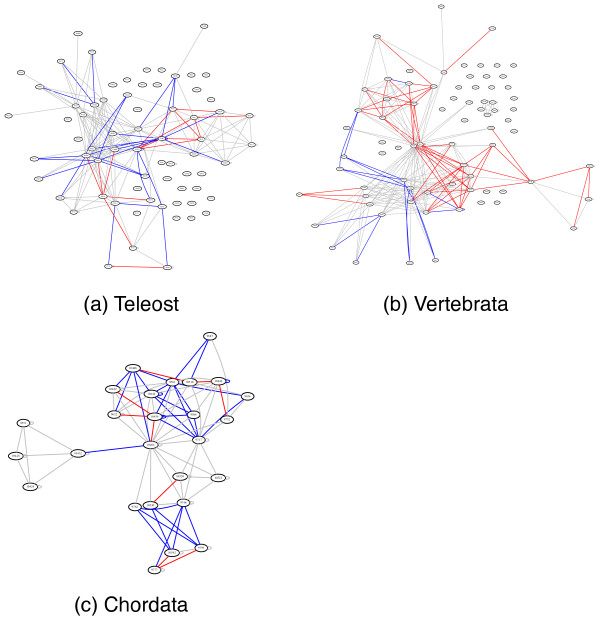
**bZIP Reconstructions.** The inferred networks of the Teleost, Vertebrata and Chordata ancestors. Edges drawn in gray were inferred by both our parsimony-based approach and by the sequence-based approach. Red edges were inferred based on sequence but not by the parsimony method, and the blue edges were inferred by the parsimony method but not based on sequence.

A visual inspection (see Figure 4) of the inferred ancestral networks revealed no strong patterns among the interactions predicted based on sequence versus those predicted using our parsimony approach. However, if a protein is involved in a disagreement, it is often involved in more than one.

## Conclusion

We have presented a novel framework for representing network histories involving gene duplications, gene loss, and interaction gain and loss for both directed and undirected graphs. We also provide a combinatorial characterization for valid histories. Our experiments demonstrate that a fast heuristic can recover optimal histories in a large majority of instances. We further provide evidence that, even with a probabilistic, weighted, generative model of network growth, a parsimony approach can recover accurate ancestral networks (F1 scores ≥ 0.8 for a wide range of parameters under several different models). Finally, we show that our method accurately reconstructs a number of ancestral networks for the bZIP family of proteins. Interestingly, we observe that we obtain the highest accuracy in ancestral network reconstruction when we do not impose a particular ordering on unrelated duplication events (as implied by phylogenetic branch lengths). This suggests that the ability of our approach to explore a larger space of potential solutions than previous work can provide practical benefits. In future work, it will be interesting to explore topological properties of the ancestral networks, such as modularity and degree distribution, and to analyze how these properties may have changed over time. We would also like to extend the evolutionary history framework and inference algorithm to handle *de novo* gene birth events, which are known to contribute to network growth [27].

## Competing interests

The authors declare that they have no competing interests.

## Authors’ contributions

The history encoding framework and algorithms were devised by RP, ES, JM, GM, SN and CK. The generalized regulatory models were devised and implemented by JM and RP. The algorithms were implemented by RP and ES. The synthetic tests were performed by RP and ES and the bZIP tests were performed by RP. The manuscript was written by RP, ES, JM, GM, SN and CK. All authors participated in the discussions. All authors read and approved the final manuscript.
